# Positive deviance for dual-method promotion among women in Uganda: study protocol for a cluster randomized controlled trial

**DOI:** 10.1186/s13063-020-4192-8

**Published:** 2020-03-17

**Authors:** Hodaka Kosugi, Akira Shibanuma, Junko Kiriya, Ken Ing Cherng Ong, Stephen Mucunguzi, Conrad Muzoora, Masamine Jimba

**Affiliations:** 1 Department of Community and Global Health, School of International Health, Graduate School of Medicine, The University of Tokyo, 7-3-1 Hongo, Bunkyo-ku, Tokyo, 113-0033 Japan; 2 Department of Community Health, Mbarara University of Science and Technology, P.O BOX 1410, Mbarara, Uganda; 3 Department of Internal Medicine, Mbarara University of Science and Technology, P.O BOX 1410, Mbarara, Uganda

**Keywords:** Positive deviance, Dual-method use, Contraception, Unintended pregnancy, Sexually transmitted infection, HIV/AIDS

## Abstract

**Background:**

Dual-method use is known as the most reliable protection against unintended pregnancies and sexually transmitted infections, including HIV. However, it is not commonly used in sub-Sharan Africa, especially among women using highly effective contraceptives. This article describes a protocol to evaluate the effect of an intervention formulated under the positive deviance approach for promoting dual-method use in Uganda.

**Methods:**

A total of 150 women will be interviewed using a structured questionnaire to find those practicing dual-method use. In-depth interviews will then be conducted with all women using the dual method and 10 women using only highly effective contraceptives to identify their unique practice. Then, a cluster randomized controlled trial will be conducted to examine the effect of an intervention formulated under the positive deviance approach on dual-method uptake and adherence. Twenty health facilities will be randomized to an intervention or control arm and 480 women will be enrolled in each group. The participants will be followed up for 8 months.

**Discussion:**

This trial focuses on women who already adapted dual-method use and identifies their unique solutions to promote dual-method use. This trial could tackle barriers for dual-method use, which expert outsiders may fail to recognize, by analyzing and promulgating their unique behaviors. This study could provide evidence that the positive deviance approach can address unintended pregnancies and sexually transmitted infections as well as other health problems which usual approaches have failed to address.

**Trial registration:**

UMIN-CTR Clinical Trial, UMIN000037065. Registered on 14 June 2019.

## Background

In sub-Saharan Africa (SSA), women of reproductive age bear a disproportionate burden of unintended pregnancies and sexually transmitted infections (STIs) including human immunodeficiency virus (HIV) [1]. In SSA, an estimated 29% of pregnancies are unintended [2]. Moreover, women account for approximately 56% of all adults living with HIV in this region [3]. This gender disparity starts when women reach their reproductive age, and women represent 59% of new HIV infections in this region [3, 4].

Unintended pregnancies occur because appropriate methods of contraception are not available or avoided [5]. To prevent this, highly effective contraceptives (HECs), such as hormonal contraceptives (e.g. pills, injectables, and implants), non-hormonal intrauterine device (IUD), and sterilization, were introduced to family planning programs [5]. In many countries in SSA, women have started to use these methods more frequently in recent decades [6]. HECs are effective in preventing unintended pregnancies but cannot prevent HIV/STIs [7]. Therefore, women need to protect themselves from HIV/STIs, regardless of whether they are using HECs or not.

Dual protection is defined as protection against the dual risks of unintended pregnancies and HIV/STIs [8]. It can be accomplished by either using a condom consistently alone or with HECs (dual-method use) [8]. Condoms are an effective method for women of preventing HIV/STIs from their sexual partners [9]. However, as they are often used incorrectly and inconsistently, condoms can only prevent 85% of pregnancies [10]. Dual-method use, thus, has been recommended as the most reliable protection against the dual risks in couples who do not want a child or who want to delay childbirth [7, 8, 11, 12]. Nevertheless, it remains uncommon [11]. In the United States, 7% of reproductive-age women who were sexually active used this method [13]. In SSA, most research has focused on dual-method use among women living with HIV and adolescents. For instance, 16% and 39% of women living with HIV practiced dual-method in 3 months in Ethiopia and Kenya, respectively [8, 14], while 7% of South African adolescents aged 15–24 years reported dual-method use [15].

A trade-off between HEC and use of condoms is a barrier to practicing dual protection. Women are less likely to use condoms with their male partners when using HECs [16]. Use of condoms may become unacceptable, especially in marital sex, as it is perceived as protection against HIV/STIs rather than pregnancies. Both women and men may think condoms are unnecessary with an intimate partner, especially when women are using HECs. However, use of condoms is necessary for women who are at risk of HIV/STIs, regardless of use of HEC [17, 18]. Extramarital sexual relationships are common, especially among men, in SSA [19]. For instance, an estimated 44% of HIV infections occurred among married or cohabiting couples in Kenya [20].

Several interventions have been conducted to promote dual-method use in the USA [11, 12]. However, few interventions had a significant effect on dual-method use [11, 12, 21] and the impact of such interventions was often not sustainable [22]. In resource-limited settings, including SSA, no interventions have been examined, although people are at considerable risk of unintended pregnancies and HIV/ STIs [11]. Women and men may perceive the importance of the use of condoms for preventing HIV/STIs, but often do not practice it [23]. Motivating factors for dual-method use remain unknown when the percentage of such users is low.

The positive deviance approach has the potential to address barriers to sensitive issues such as sexual and reproductive health. This approach seeks behaviors that contribute to otherwise high-risk individuals, or positive deviants, remaining free from a disease or condition and enables communities to adopt such practices [24, 25]. This approach has addressed complex development challenges, which are often hard for expert outsiders to measure, such as gender-related and socio-cultural barriers [24]. For example, the positive deviance approach was applied to advocate against female genital mutilation using actual words of positive deviants in Egypt [24]. Use of condoms is not prevalent in SSA, especially among married women using HECs. Barriers to condom use are complex and it is often difficult for outsiders to grasp the whole picture [26]. Given the limited effect of previous interventions [11], the positive deviance approach could be an ideal option for promoting dual-method use.

The present article illustrates a study protocol to examine the effect of an intervention formulated under the positive deviance approach on dual-method use among married women using HECs with their partners in Uganda.

## Methods

### Study setting

The present study is conducted in Mbarara district, south-western Uganda. Use of contraceptives has significantly increased in Uganda. Its use among married women increased from 14% in 2001 to 35% in 2014 [27]. Like other countries in SSA, HECs are becoming the norm in Uganda, with 32% of currently married women using them in 2014 [27]. Injectables are the most used method (19%) followed by implants (6%), female sterilization (3%), pills (2%), and IUD (2%) [27]. Despite the significant increase in the use of contraceptives, an estimated 46% of pregnancies were unintended in Uganda in 2018 [28]. The prevalence of HIV among adults aged 15–64 years is 6.2% and is higher among women (7.6%) than among men (4.7%) [29]. The south-west region had the second highest prevalence of HIV (7.9%) after the Central region (8.0%) in Uganda [29].

Mbarara district has one regional hospital, six general hospitals, four county-level health centers (health center IV), 14 sub-county-level health centers (health center III), and 37 parish-level health centers (health center II) [30]. Among them, 48 are public health facilities and 23 facilities are located in urban areas [30]. A family planning service is provided for free at all levels of health centers. Male and female condoms are also provided free by the Ministry of Health and by local and international non-governmental organizations [31]. Condoms can also be purchased from supermarkets and pharmacies for US$0.15–US$0.50 [31].

### Study objectives

The objectives of the present study are: (1) to identify unique behaviors that are common only among married women who practice dual-method use with their partners in an HIV-prevalent setting in Uganda; and (2) to evaluate the effect of an intervention formulated under the positive deviance approach for promoting dual-method use among married women using HECs.

### Study design

**Fig. 1:**
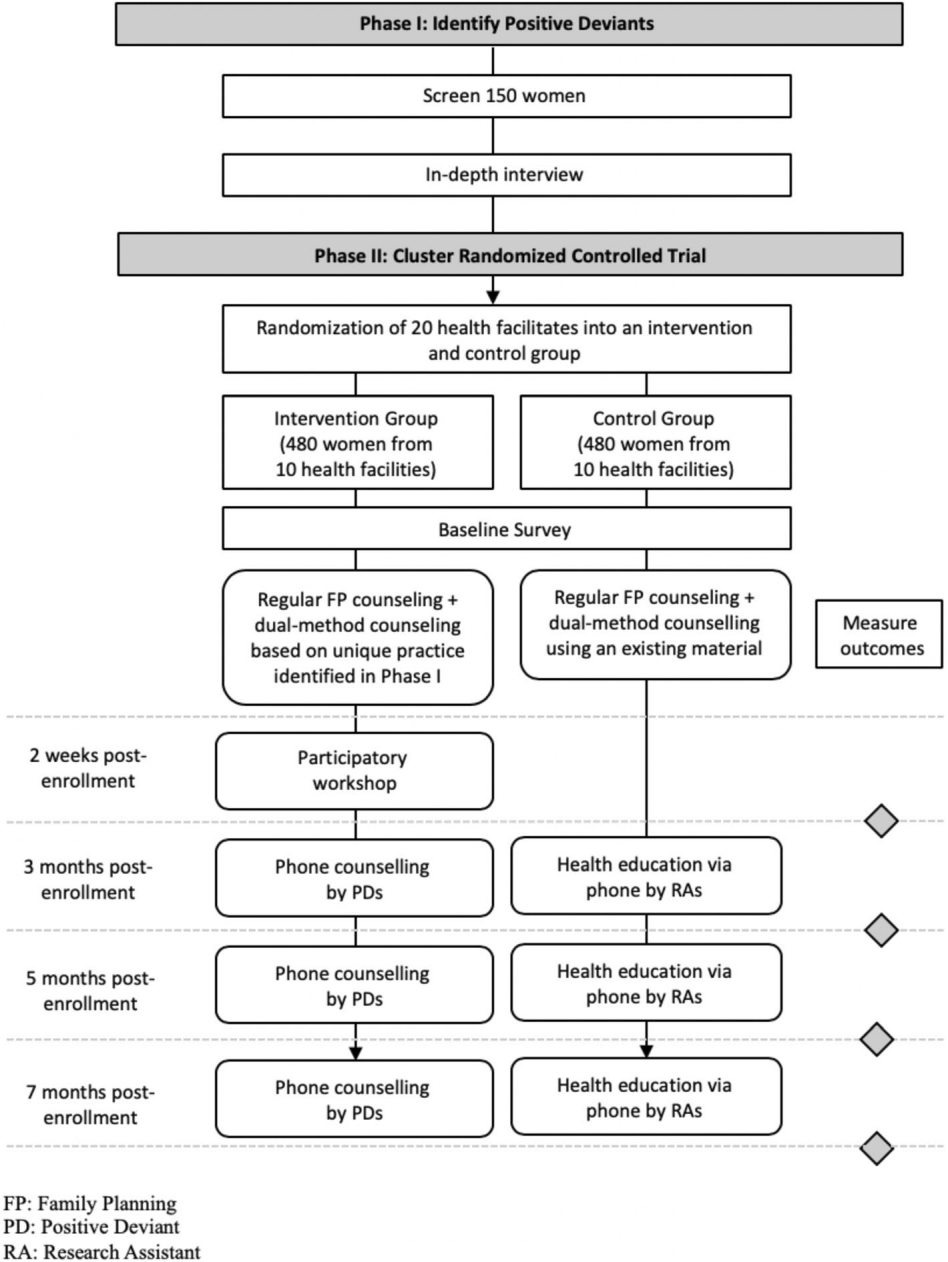
Study flow chart

**Fig. 2:**
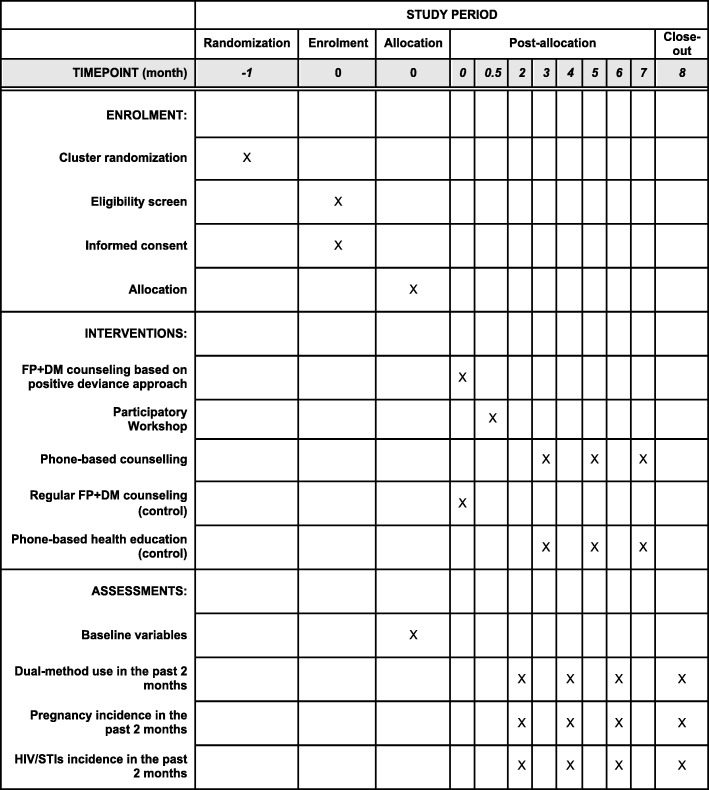
SPIRIT figure

The present study consists of Phases I and II. In Phase I, eligible women will be screened with a structured questionnaire. In-depth interviews will then be conducted with all women practicing dual-method use and 10 women using only HECs to identify the unique practice that is common only among women practicing dual-method use (positive deviants). In Phase II, a cluster randomized controlled trial (C-RCT) will be conducted to assess an intervention formulated under the positive deviance approach for promoting dual-method use. The intervention will consist of clinic-based and phone counseling and a participatory workshop that will be tailored based on the unique practice identified in Phase I. The overall study flow chart is shown in Fig. 1. The schedule of the C-RCT is shown in the Standard Protocol Items: Recommendations for Intervention Trials (SPIRIT) figure (Fig. 2). The SPIRIT checklist is provided in Additional file 1.

#### Phase I: identify positive deviants

##### Sample size, eligibility, and recruitment

In Phase I, 150 women will be interviewed. This is based on the assumption that at least 7% of women would practice dual-method use [13] and at least 10 women who are considered as a positive deviant would be identified.

To be eligible for joining this study, participants should have the following characteristics: (1) women; (2) aged 18–49 years; (3) sexually active; (4) using HECs at the time of recruitment; (5) have a desire to avoid pregnancy for 12 months from recruitment; (6) have a husband or live-in sexual partner; and (7) have access to a valid phone number.

Being sexually active is defined as having had sexual intercourse in the 3 months before the study [13]. Pregnant women and women who are infertile for other reasons will be excluded from this study. Health workers, including community health workers, political and religious leaders, and teachers will also be excluded because they may not represent communities or be influenced by their occupations and social status.

For sampling, five health facilities will be selected purposively. Then, five trained female research assistants will approach female clients in the family planning sections of the selected health facilities. The first client will be selected randomly at each clinic and then every third client will be informed about the opportunity to participate in this study. If they are interested in participating, the research assistants will ask screening questions using a pretested questionnaire to check their eligibility for the study. This process will be repeated until the required sample size is met.

##### Data collection

The five female trained research assistants will conduct face-to-face interviews using a pretested structured questionnaire with the participants. These interviews aim to identify women practicing dual-method use and their basic sociodemographic characteristics. Data collection items include basic sociodemographic characteristics, the types of HECs, the frequency of condom use in the past 2 months, and the histories of unintended pregnancies and diagnosed HIV/STIs. Women using both HECs and condom always will be regarded as practicing dual-method use. Dual-method users without no reported histories of unintended pregnancies and HIV/STIs will be considered as positive deviants.

Next, in-depth interviews will be conducted by the research assistants with all positive deviants to identify their unique practice, such as effective communication for use of condoms that is actually working. Then, 10 women who do not practice dual-method use will be randomly selected for in-depth interviews to document common practices and verify if the practice identified in positive deviants is unique. The interviews will be open-ended and an interview guide will be used. The interview guide focuses on the following domains: (1) perceptions of condom use and contraception; (2) reasons and motivations for condom use or non-use; (3) negotiation and communication for condom use; and (4) risk perceptions for or unintended pregnancy and HIV/STIs.

The data collection tools were first developed in English and then translated to Runyankore by an independent researcher. They were back-translated to English by the Ugandan researcher (SM) to ensure the accuracy of the translation. All interviews will be conducted in either English or Runyankore. In-depth interviews will be audio recorded based on the consent of participants. All participants will be given some commodities worth 10,000 Ugandan Shillings (UGX) (equivalent to US$3) after the initial interview; those who participate in the in-depth interview will receive 10,000 UGX for their time and transportation.

##### Data analysis plan

All qualitative interview data will be transcribed, and if not in English, translated from Runyankore into English by a research assistant. Translated transcriptions will be compared with recorded data by the researcher (SM) to ensure their accuracy. Then, the two researchers (HK, SM) and the five research assistants who conducted in-depth interviews will read all the transcripts and code overarching themes using MAXQDA version 18. Then, data will be compared between dual-method users and non-users to identify problems and barriers to adapting dual-method use and how they were overcome by positive deviants with their unique practice.

#### Phase II: cluster randomized controlled trial

##### Sample, eligibility, and recruitment

The minimum required sample size for this trial is 588. It was calculated based on data from research on the effect of case management and peer education program on the uptake of dual- method use in the USA (odds ratio [OR] = 1.58, 95% confidence interval [CI] = 1.03–2.43) [32]. The less conservative effect size of 2.43 was used because this trial will include only women who have already used HECs, unlike the prior research which recruited adolescent girls regardless of their contraceptive use [32]. Then, an intraclass correlation coefficient (ICC) of 0.006 was considered [33, 34]. The ICC was adapted from a clinic-based condom use intervention in SSA [33]. The required minimum sample size was 760 after considering the ICC. Considering a 26% dropout rate, 960 participants will be recruited (480 participants in each arm) [22]. The power of the study was set at 80% and the significance level was set at 5%. Open Epi version 3 was used for the calculation.

Twenty health facilities will be purposively selected considering the size and rural/urban status. The same inclusion criteria as Phase I will be used for this intervention study, but if women have been practicing dual-method use in the 2 months before recruitment, they will be excluded. The same sampling method as Phase I will be used to recruit women at health facilities. Eighty women will be recruited from each of the hospitals and county-level health centers, and 40 from each of the sub-county-level health centers and parish-level health centers.

##### Randomization

To minimize contamination across individuals, the C-RCT approach will be adopted [35]. The 20 health facilities will be stratified based on the level of health facilities and urban/rural status and randomized to the intervention (n = 10 facilities) or control arm (n = 10 facilities) (1:1 allocation ratio), using a computer random number generator by the principal investigator (HK). The participants will be given the intervention that the facilities they were recruited at were allocated to.

##### Intervention

The intervention will consist of a series of counseling and participatory workshops, planned based on the unique practice identified in Phase I. On the day of enrollment, women in the intervention arm will receive dual-method counseling with a tool developed based on the practice identified in Phase I, in addition to regular family planning counseling with an existing counseling tool [36]. The dual-method counseling will be conducted for 20–40 min by trained research assistants.

Two weeks after enrollment, women in the intervention arm will be invited for a 1-day participatory learning workshop (5 h) at the same health facilities where they were recruited. Women may decide whether to participate. The workshop will be facilitated by research assistants and positive deviants, using a training protocol developed after Phase I. It will include simulations and role-plays for successful communication about using a condom with their partners and a group discussion regarding family planning and HIV/STI risk.

Bi-monthly telephone counseling will be provided by positive deviants three times (3, 5, and 7 months after enrollment). Each counseling session will take 10–20 min and aim to remind women of the risk of unintended pregnancies and HIV/STIs and strengthen their capacity to communicate using a condom with their partners. The 2-month intervals can allow women to reflect on counseling messages, discuss the use of condoms with their partners, and put it into practice. Positive deviants, therefore, can provide more effective counseling tailored to participants’ individual needs through understanding their situation.

Women in the control group will be provided regular family planning counseling including dual-method use for 10–30 min by trained research assistants using the same existing tool on the day of enrollment [36]. Moreover, refresher training will be provided bi-monthly on family planning and HIV/STI risk via phone, by research assistants three times (3, 5, and 7 months after the enrollment).

Condoms will be provided for free, regardless of the allocation at the selected health facilities. Before the intervention starts, the research assistants will receive a 2-day training session about the contents of the existing counseling tool by health professionals [36].

##### Outcomes

The primary outcome is dual-method use. In this study, dual-method use is defined as the use of male or female condoms along with HECs such as pills, injectables, implants, male and female sterilization, and IUD consistently in the last 2 months, before each follow-up interview [8]. The outcome measure combines two questions regarding the frequency of condom use and HEC use. The frequency of condom use will be asked with an item: “How often did you and your partner use a male or female condom during the past 2 months?” Women will answer this question using a four-point scale “every time,” “almost every time,” “sometimes,” and “never.” Only those who answered “every time” will be considered as having consistent condom use. Women will also be asked if they have been using any HECs with a question, “Apart from condoms, have you been using any other forms of protection against pregnancy during the past two months?” Responses to these two questions will be used to construct the dual-method use outcome with the following categories: (1) dual-method use (consistent condom and HEC use); (2) inconsistent use of condoms and HECs; and (3) single or no use of contraceptive methods.

The secondary outcome is the self-reported first occurrence of pregnancy and STIs in the previous 2 months [37]. They will be assessed with the following two questions: “Have you been told by a healthcare provider that you got pregnant for the first time in the past 2 months?” and “Have you been told by a healthcare provider that you had any STIs such as chlamydia, gonorrhea, or trichomonas infection for the first time in the past 2 months?”

##### Other information

The following information will be collected during the baseline interviews to conduct sub-group analyses and to identify factors associated with the use of condoms: age; education; employment; area of residence (rural or urban); reproductive history; pregnancy intention; sexual history; STI history; substance use; domestic violence; contraceptive methods in use; awareness of dual-method use; spousal communication on family planning; HIV status of participants and their partners; disclosure of HIV status; antiretroviral therapy (ART) treatment status [14, 22, 38]; HIV-related knowledge (HIV-KQ-18) [39]; perceived STI and HIV infection risk [40]; condom use self-efficacy [41]; sexual relationship control power (the Sexual Relationship Power Scale [SRPS]) [42]; and women’s perception of the social acceptability of contraception [43].

##### Data collection

On the day of enrollment, all women will be interviewed using a pretested structured questionnaire to identify basic baseline characteristics. They will then be followed up via phone bi-monthly for 8 months to assess how the intervention influences dual-method use and pregnancy and STI incidence (2, 4, 6, and 8 months after enrollment). All interviews will be conducted by trained female research assistants using an interview guide in either English or Runyankore. All data will be entered using EpiData version 4.6.

All participants will be given some incentives worth 10,000 UGX (equivalent to US$3) for their time and participation in the study after the initial interviews. Participants who participate in the learning workshop will receive 20,000 UGX (equivalent to US$6) for transportation.

##### Data analysis

The background characteristics of women will be compared between the intervention and the control group using Pearson’s chi-squared or Fisher’s exact tests [44]. Mixed effects logistic regression analysis will be performed with a random effects term for the clusters to access the effect of the intervention on the following outcomes: dual-method selection and adherence; self-reporting pregnancy; and incidence of STI in the 2 months before each follow-up data collection. In addition, hierarchical logistic regression model will be introduced to ascertain the predictors of each outcome. Model 1 will be adjusted for demographic variables, such as age, education, and the number of children. Model 2 will be adjusted for the status of residence (urban or rural) in addition to demographic variables. Model 3 will include the type of HECs at the baseline sequentially, which is known to be associated with dual-method use [8, 45]. Model 4 will be adjusted for variables related to their partners, such as partner’s attitude toward family planning. Model 5 will include women’s perceived risk of HIV/STIs. Model 6 (full model) will be adjusted for all variables. Besides, sub-group analyses will be conducted among HIV-seroconcordant and -discordant couples. All the analyses will be conducted on both per-protocol and intention-to-treat bases. The per-protocol analysis will assess the efficacy of the intervention while the intention-to-treat analysis will test its effectiveness [46]. Rates of attrition and reasons for dropout will be compared between the intervention and the control groups [47]. The significance level will be set at 5%. STATA version 13.1 will be used for all data analyses.

## Discussion

The present study will be a unique trial to examine the effect of the intervention to promote dual-method use among women using the positive deviance approach. Dual-method use can reduce unintended pregnancies and HIV/STIs among women of reproductive age. Use of condoms is necessary for dual protection but is not commonly practiced in SSA, especially among married couples [9].

The present trial could tackle barriers to dual-method use, which expert outsiders may fail to recognize, by taking the positive deviance approach. This approach could be an ideal option when a problem needs behavioral change but is not merely technical [48]. The present study focuses on women who have already adapted the dual method and identifies unique effective communication and behavioral strategies that are actually working. Their unique solutions are analyzed and promulgated to other women who do not practice dual-method use. Unlike the usual approach, which looks for solutions from the outside, this approach could overcome barriers to dual-method use and bring sustainable effects by adopting local solutions. The present trial aims to provide evidence that the positive deviance approach can tackle not only dual-method use but also other issues that usual approaches have failed to address, by making use of local wisdom and solutions in Uganda.

The present study has several limitations. First, the success of the positive deviance approach depends on whether women practicing dual-method use or positive deviants can be found in the study area [24, 48]. Considering the prevalence of such people, the sample size can be too small and may need to be modified. Second, positive deviants are identified by the research team not by community members in this trial. Therefore, the effect of the intervention can be limited in mobilizing and empowering communities to adopt identified strategies, compared to the usual approach including self-discovery of positive deviants [24, 48]. Third, high attrition is anticipated, especially among women in the control arm, as all the interventions and follow-ups after recruitment and initial counseling are phone-based [11]. The present trial, therefore, calculated the sample size considering a relatively high attrition rate to offset potential losses to follow-up. Moreover, it will be minimized by building rapport with participants. For this, each participant will be contacted by the same positive deviant and/or research assistant for counseling and follow-up throughout the study period. Fourth, the outcomes will be assessed based on self-reports from participants and thus subject to response bias. Dual-method use can be over-reported while the incidence of STIs and unintended pregnancies can be under-reported due to social desirability bias. However, response bias will be minimized by assuring each participant of confidentiality of responses at the enrollment. Lastly, findings of the present study might lack external validity to other locations as it will focus on one particular community under the positive deviance approach [48]. In the present study, participants will be recruited in only public health facilities; thus, findings from this study may not be generalized to all women using HECs. To apply findings and the interventions developed in the present study to other areas and populations, further research will be necessary to assess its effectiveness in a given context.

The proposed research is designed to add evidence to the effectiveness of the positive deviance approach in tackling problems that the usual approach has failed to address, such as dual-method use. The results will be useful for public health policymakers to develop programs to reach women who need to tackle unintended pregnancies and HIV/STIs as well as other health problems that other solutions fail to address.

### Trial status

Enrollment into the trial is expected to start in October 2019 and end in November 2019. The trial is expected to be completed by June 2020. This manuscript was based on the study protocol version 8 and the version date is 5 August 2019.

## Supplementary information

**Additional file 1.** SPIRIT 2013 Checklist: Positive deviance for dual-method promotion among women in Uganda: Study protocol for a cluster randomized controlled trial.

## Data Availability

The data collection tools and datasets generated during the study will be available from the corresponding author upon request following the publication of results.

## Abbreviations

ART: Antiretroviral therapy
C-RCT: Cluster randomized controlled trial
FP: Family planning
HEC: Highly effective contraceptive
HIV: Human immunodeficiency virus
ICC: Intraclass correlation coefficient
IUD: Intrauterine device
PD: Positive deviant
RA: Research assistant
SPIRIT: Standard Protocol Items: Recommendations for Intervention Trials
SRPS: Sexual Relationship Power Scale
SSA: Sub-Saharan Africa
STIs: Sexually transmitted infections
UGX: Ugandan Shilling
USA: United States of America
USD: United States Dollar

## References

[1] Karim SA, Baxter C, Frohlich J, Karim QA (2014). The need for multipurpose prevention technologies in sub-Saharan Africa. BJOG..

[2] Ameyaw EK, Budu E, Sambah F, Baatiema L, Appiah F, Seidu AA (2019). Prevalence and determinants of unintended pregnancy in sub-Saharan Africa: A multi-country analysis of demographic and health surveys. PLoS One.

[3] Joint United Nations Programme on HIV and AIDS (UNAIDS). UNAIDS data 2018. Geneva: UNAIDS; 2018. https://www.unaids.org/sites/default/files/media_asset/unaids-data-2018_en.pdf. Accessed 7 Sept 2019.

[4] Kharsany AB, Karim QA (2016). HIV infection and AIDS in sub-Saharan Africa: current status, challenges and opportunities. Open AIDS J.

[5] Hubacher D, Mavranezouli I, McGinn E (2008). Unintended pregnancy in sub-Saharan Africa: magnitude of the problem and potential role of contraceptive implants to alleviate it. Contraception.

[6] United Nations, Department of Economic and Social Affairs, Population Division. Estimates and projections of family planning indicators 2019. New York: United Nations; 2019. https://www.un.org/en/development/desa/population/publications/pdf/family/Figure_Model-based_estimates_Countries_2019.pdf. Accessed 7 Sept 2019.

[7] Pazol K, Kramer MR, Hogue CJ (2010). Condoms for dual protection: patterns of use with highly effective contraceptive methods. Public Health Rep.

[8] Gebrehiwot SW, Azeze GA, Robles CC, Adinew YM (2017). Utilization of dual contraception method among reproductive age women on antiretroviral therapy in selected public hospitals of Northern Ethiopia. Reprod Health.

[9] Maticka-Tyndale E (2012). Condoms in sub-Saharan Africa. Sex Health.

[10] Kraft JM, Galavotti C, Carter M, Jamieson DJ, Busang L, Fleming D (2009). Use of dual protection in Botswana. Stud Fam Plan.

[11] Lopez LM, Stockton LL, Chen M, Steiner MJ, Gallo MF (2014). Behavioral interventions for improving dual-method contraceptive use. Cochrane Database Syst Rev.

[12] O'Leary A (2011). Are dual-method messages undermining STI/HIV prevention?. Infect Dis Obstet Gynecol.

[13] Eisenberg DL, Allsworth JE, Zhao Q, Peipert JF (2012). Correlates of dual-method contraceptive use: an analysis of the National Survey Of Family Growth (2006-2008). Infect Dis Obstet Gynecol.

[14] Mulongo AM, Lihana RW, Githuku J, Gura Z, Karanja S (2017). Factors associated with uptake of dual contraception among HIV-infected women in Bungoma County, Kenya: a cross-sectional study. Pan Afr Med J.

[15] MacPhail C, Pettifor A, Pascoe S, Rees H (2007). Predictors of dual method use for pregnancy and HIV prevention among adolescent South African women. Contraception.

[16] Ott MA, Adler NE, Millstein SG, Tschann JM, Ellen JM (2002). The trade-off between hormonal contraceptives and condoms among adolescents. Perspect Sex Reprod Health.

[17] Kosugi H, Shibanuma A, Kiriya J, Wafula SW, Jimba M (2019). Consistent condom use among highly effective contraceptive users in an HIV-endemic area in rural Kenya. PLoS One.

[18] Williamson NE, Liku J, McLoughlin K, Nyamongo IK, Nakayima F (2006). A qualitative study of condom use among married couples in Kampala, Uganda. Reprod Health Matters.

[19] Ramjee G, Daniels B (2013). Women and HIV in sub-Saharan Africa. AIDS Res Ther.

[20] National Aids Control Council (NACC). Kenya HIV estimates report, 2015. Nairobi: NACC; 2016. http://nacc.or.ke/wp-content/uploads/2016/12/Kenya-HIV-Estimates-2015.pdf. Accessed 7 Sept 2019.

[21] El Ayadi AM, Rocca CH, Kohn JE, Velazquez D, Blum M, Newmann SJ (2017). The impact of an IUD and implant intervention on dual method use among young women: Results from a cluster randomized trial. Prev Med.

[22] Peipert JF, Zhao Q, Meints L, Peipert BJ, Redding CA, Allsworth JE (2011). Adherence to dual-method contraceptive use. Contraception.

[23] Woodsong C, Koo HP (1999). Two good reasons: Women’s and men’s perspectives on dual contraceptive use. Soc Sci Med.

[24] Marsh DR, Schroeder DG, Dearden KA, Sternin J, Sternin M (2004). The power of positive deviance. BMJ..

[25] Singhal A, Svenkerud PJ (2018). Diffusion of evidence-based interventions or practice-based positive deviations. J Dev Commun.

[26] Mutowo J, Kasu C (2015). Barriers to use of dual protection among married women in a suburban setting. IOSR-JNHS.

[27] Uganda Bureau of Statistics (2018). Uganda demographic and health survey 2016.

[28] Performance Monitoring and Accountability 2020 (PMA2020). PMA2020/Uganda: April–May 2018 (round 6). PMA2020. 2018. https://www.pma2020.org/sites/default/files/PMA2020-Uganda-R6-FP-brief.pdf. Accessed 12 Dec 2019.

[29] Ministry of Health (MOH) (2019). Uganda population-based HIV impact assessment: (UPHIA) 2016–2017.

[30] Ministry of Health (MOH) (2018). National health facility master list 2018.

[31] Makerere University, School of Public Health (2015). Rapid assessment of comprehensive condom programming in Uganda.

[32] Sieving RE, McMorris BJ, Beckman KJ, Pettingell SL, Secor-Turner M, Kugler K (2011). Prime time: 12-month sexual health outcomes of a clinic-based intervention to prevent pregnancy risk behaviors. J Adolesc Health.

[33] Zhang J, Pals SL, Medley A, Nichols C, Bachanas P, van Zyl D (2014). Parameters for sample size estimation from a group-randomized HIV prevention trial in HIV clinics in sub-Saharan Africa. AIDS Behav.

[34] van Breukelen GJ, Candel MJ (2012). Calculating sample sizes for cluster randomized trials: we can keep it simple and efficient!. J Clin Epidemiol.

[35] Hemming K, Eldridge S, Forbes G, Weijer C, Taljaard M (2017). How to design efficient cluster randomised trials. BMJ.

[36] World Health Organization (WHO) (2005). Decision-making tool for family planning clients and providers.

[37] Ewing AC, Kottke MJ, Kraft JM, Sales JM, Brown JL, Goedken P (2017). 2GETHER - The dual protection project: design and rationale of a randomized controlled trial to increase dual protection strategy selection and adherence among African American adolescent females. Contemp Clin Trials.

[38] Antelman G, Medley A, Mbatia R, Pals S, Arthur G, Haberlen S (2015). Pregnancy desire and dual method contraceptive use among people living with HIV attending clinical care in Kenya, Namibia and Tanzania. J Fam Plann Reprod Health Care.

[39] Carey MP, Schroder KE (2002). Development and psychometric evaluation of the brief HIV knowledge questionnaire. AIDS Educ Prev.

[40] Napper LE, Fisher DG, Reynolds GL (2012). Development of the perceived risk of HIV scale. AIDS Behav.

[41] Shaweno D, Tekletsadik E (2013). Validation of the condom use self-efficacy scale in Ethiopia. BMC Int Health Hum Rights.

[42] Pulerwitz J, Gortmaker SL, DeJong W (2000). Measuring sexual relationship power in HIV/STD research. Sex Roles.

[43] Samandari G, Speizer IS, O’Connell K (2010). The role of social support and parity in contraceptive use in Cambodia. Int Perspect Sex Reprod Health.

[44] Twisk J, Bosman L, Hoekstra T, Rijnhart J, Welten M, Heymans M (2018). Different ways to estimate treatment effects in randomised controlled trials. Contemp Clin Trials Commun.

[45] Steiner RJ, Liddon N, Swartzendruber AL, Rasberry CN, Sales JM (2016). Long-acting reversible contraception and condom use among female US high school students: Implications for sexually transmitted infection prevention. JAMA Pediatr.

[46] Shah PB (2011). Intention-to-treat and per-protocol analysis. CMAJ.

[47] Kolamunnage-Dona R, Powell C, Williamson PR (2016). Modelling variable dropout in randomised controlled trials with longitudinal outcomes: application to the MAGNETIC study. Trials..

[48] Lapping K, Marsh DR, Rosenbaum J, Swedberg E, Sternin J, Sternin M (2002). The positive deviance approach: challenges and opportunities for the future. Food Nutr Bull.

